# The Feedback-Related Negativity and the P300 Brain Potential Are Sensitive to Price Expectation Violations in a Virtual Shopping Task

**DOI:** 10.1371/journal.pone.0163150

**Published:** 2016-09-22

**Authors:** Alexandre Schaefer, Luciano G. Buratto, Nobuhiko Goto, Emilie V. Brotherhood

**Affiliations:** 1 Durham University, Department of Psychology, Durham, United Kingdom; 2 Monash University, Malaysia Campus, Department of Psychology, Bandar Sunway, Malaysia; 3 Monash University, Malaysia Campus, School of Business, Bandar Sunway, Malaysia; 4 University of Brasília, Institute of Psychology, Brasília, Brazil; 5 University College London, Institute of Neurology, London, United Kingdom; University of Vienna, AUSTRIA

## Abstract

A large body of evidence shows that buying behaviour is strongly determined by consumers’ price expectations and the extent to which real prices violate these expectations. Despite the importance of this phenomenon, little is known regarding its neural mechanisms. Here we show that two patterns of electrical brain activity known to index prediction errors–the Feedback-Related Negativity (FRN) and the feedback-related P300 –were sensitive to price offers that were cheaper than participants’ expectations. In addition, we also found that FRN amplitude time-locked to price offers predicted whether a product would be subsequently purchased or not, and further analyses suggest that this result was driven by the sensitivity of the FRN to positive price expectation violations. This finding strongly suggests that ensembles of neurons coding positive prediction errors play a critical role in real-life consumer behaviour. Further, these findings indicate that theoretical models based on the notion of prediction error, such as the Reinforcement Learning Theory, can provide a neurobiologically grounded account of consumer behavior.

## Introduction

It is often assumed that consumers form mental representations of a product’s market price through prior encounters with products and their prices [1,2]. Discrepancies between these expectations and actual prices are known to bias purchasing decisions and, in particular, positive discrepancies (when actual prices are cheaper than expected) play an important role in facilitating the action of purchasing goods [1]. Although the importance of this process for the wider economy is obvious, its neurobiological mechanisms have yet to be fully understood, although recent advances in consumer neuroscience research are very promising [3–8].

From the point of view of cognitive neuroscience, discrepancies between learned predictions and actual events have often been modeled using reinforcement learning (RL) theory. The basic formulation of RL models is that the brain forms predictions about future events through learning from prior instances of positive and negative reinforcements [9]. When an event deviates from prior predictions, then a *prediction error* (PE) is detected. Prediction errors can be positive (when the event is better than expected) or negative (when the event is worse than expected) and can be used to adjust future predictions and bias decisions [9]. It is thought that PEs are linked to changes in dopamine firing rates originating from a number of subcortical structures including the ventral tegmental area (VTA), which in turn send prediction error signals that modulate neurons in the Anterior Cingulate Cortex (ACC), a brain structure centrally involved in decision-making behaviour [10,11]. This framework can be applied to consumer behaviour: Positive discrepancies between expected and actual prices (when prices are cheaper than expected) can be translated into positive prediction errors (PPEs) and negative discrepancies (when prices are more expensive than expected) into negative prediction errors (NPEs).

From this perspective, we hypothesized that neural systems coding prediction errors would be strongly involved in price evaluation behaviours in a shopping context. Specifically, we hypothesized that price expectation violations would be linked to brain activity related to the detection of prediction errors. Previous research using functional magnetic resonance imaging (fMRI) has reported a link between activity in the medial prefrontal cortex (MPFC) and pricing effects [3,7]. Although the MPFC is very likely to be involved in the monitoring of prediction errors [12], it has also been linked to a number of other functions [13–17], and thus a neural signal that specifically indexes PEs would be needed to fully test the hypothesis of a link between pricing effects and neural processes of error monitoring.

**Fig 1:**
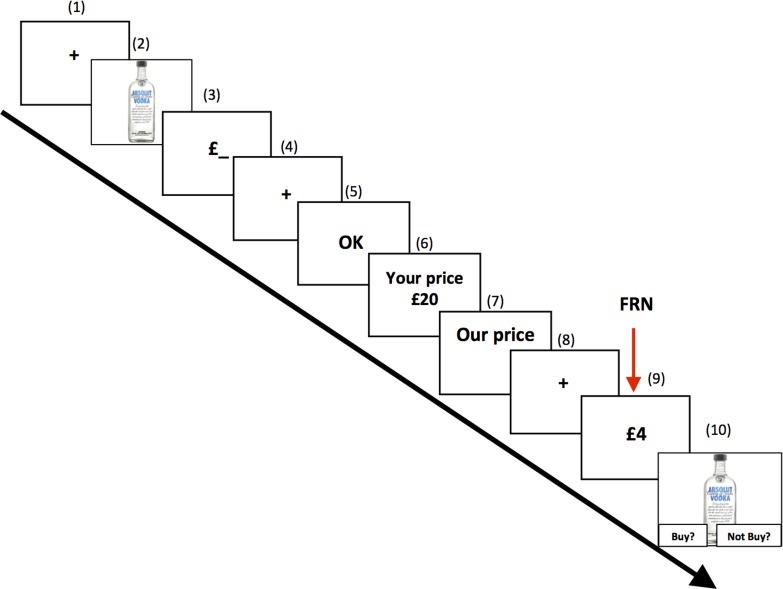
Trial procedure. (1) A fixation screen was displayed for a random duration (800 to 1,700 ms); (2) A picture of the product was presented for 2 seconds, after which a brief written description of the product was superimposed on the picture for 2 seconds; (3) Participants were prompted to estimate the price of the product and input it via the keyboard; (4) A fixation screen was displayed (800–1,700 ms, random duration); (5) A screen displayed a feedback on whether the participant’s estimation was within an acceptable range around the average market price of the product. If the estimate price was above or below 60% of the actual market price, then a “Too high” or “Too low” feedback was displayed for 1 second. In this case, the current trial was aborted and the next trial would start immediately. Otherwise, an “OK” sign was displayed and the trial could continue until its end. This approach was adopted to minimize strategic underestimations, as skipping stages 6–10 of the current trial removed the participant’s chance of buying the current product. An average of 10% of trials were skipped, and the percentage of skipped trials did not significantly differ between conditions. (6) Participants’ expected price (provided in stage 2) was displayed during 1 second; (7) The message “Our Price” was displayed for 750 ms; (8) A fixation screen was displayed (800–1,700 ms); (9) Participants were shown the actual “offer price” of the product during 1,500 ms; (10) Participants were asked to decide to buy or not the product via a key press.

We report here a study which is to our knowledge the first to show that the Feedback-Related Negativity (FRN), a well-known neural index of prediction error [10–12,18,19], is sensitive to price expectation violations during a realistic shopping situation. In this study, we asked a sample of students from a British university to perform a virtual shopping task while their electroencephalogram (EEG) was recorded. This task involved watching a series of products on a screen and estimating their average price. After providing their estimate, participants were offered to buy or not each product with a virtual allocation of £35 (reset for every product) at an offer price set by a computer program. In half of the trials, the offer price was set to deviate on average by 8% from the participants’ estimate in order to induce a *small* prediction error. In the other half of the trials, a drastic deviation of 75% from the estimated price was used to induce a *large* prediction error. These large or small prediction errors could be positive (cheaper than estimates) or negative (more expensive than estimates), resulting in four experimental conditions: Underpriced-Large (UL), Underpriced-Small (US), Overpriced-Large (OL) and Overpriced-Small (OS). A detailed description of the task can be seen in Fig 1. At the end of the experiment, a computer program randomly selected one of the products for which a “buy” decision had been made. Consistent with previous research [3,7], the selected product was later made available to the participant, and cash “savings” corresponding to the initial allocation (£35) minus the price of the chosen product was paid to the participant.

This procedure enabled us to calculate the FRN, a brain event-related potential (ERP) first reported by Miltner et al. (1997) [20] and seen by many as a neural index of prediction errors. The FRN is characterized by a fronto-central negative deflection occurring at approximately 200–350 ms after the delivery of decision outcomes. Typically, this deflection is more negative-going for nonreward compared to reward feedbacks in decision-making tasks [10,21–24] to such an extent that the FRN has often been seen as an index of feedback valence [19]. Furthermore, the FRN seems to be generated by MPFC activity [25,26], and more particularly the dorsal ACC [25], which seems to overlap with a region called "anterior middle cingulate cortex" according to a recent view of the organization of the cingulate cortex [27]. Importantly, the FRN seems to be sensitive to variations in prediction errors: The first major theoretical account of the FRN was provided by the “Reinforcement Learning Error Related Negativity” (RL-ERN) model [11], according to which the FRN was mostly sensitive to negative prediction errors. Specifically, this model predicted that the FRN was more negative-going for decision outcomes that were “worse than expected”, and this effect was thought to account for the ability of the FRN to distinguish between reward and nonreward feedbacks. However, accumulating evidence has shown that the FRN was actually sensitive to positive feedbacks, and in particular to feedbacks that were “better than expected” [10]. Consequently, the RL-ERN model has been updated and now it contends that the FRN can be understood as a “Reward Positivity” signal [28–30]. Specifically, this new account proposes that unexpected rewards trigger a phasic increase in dopaminergic activity, leading to an inhibition of ACC neurons. This inhibition of ACC neurons would be reflected on surface electrodes as a reduction of the negative deflection typically observed in the FRN time window. Consequently, FRN amplitudes would then be more positive for unexpected rewards compared to expected rewards or nonrewards [18,28,30–33]. This interpretation seems to fit much of the published research on the FRN, as the meta-analysis of Walsh et al. (2012) indicated that more studies reported a sensitivity of the FRN to PPEs than NPEs. However, there are studies that do not fit this revised RL-ERN account. For instance, a group of studies have reported that FRN waveforms are more negative for prediction errors regardless of feedback valence [34,35]. These studies suggest that the FRN may reflect a valence-independent “salience” effect. Alternatively, these results can also be interpreted in light of models suggesting that ACC activity increases when likely outcomes fail to occur, regardless of outcome valence [12,36]. The origin of the discrepancy between these accounts and the RL-ERN account is still unclear but it might be caused by the existence of distinct groups of neurons within the ACC that seem to react in opposite ways to positive surprise [37]. Nevertheless, it has to be acknowledged that the current state of the FRN literature indicates that a vast number of published studies seem to confirm the revised RL-ERN model. In particular, the sensitivity of the FRN to positively valenced feedbacks has proven to be reliable across many studies and it is now considered as a biomarker of reward processing in clinical contexts [30].

In summary, the primary goal of this study was to test whether the FRN was sensitive to price expectation violations in a realistic shopping context. In line with the literature described above, we predicted that the polarity of FRN voltage time-locked to offer prices would be more positive for UL compared to other trial types. In addition, to allow comparisons with previous research, we also examined if the P300 was sensitive to price deviations. The P300 is a classical ERP typically related to attention [38,39] and it has also been linked to the evaluation of prediction errors in decision-making tasks [40]. As a secondary goal, we also examined whether FRN and P300 waveforms time-locked to price offers could predict subsequent buying decisions. Given that PPEs are thought to be linked to “Buy” decisions, we also expected more positive FRN amplitudes for Buy compared to No-Buy decisions.

## Methods

### Participants

Forty healthy adults from the student population of Durham University (UK) took part in this experiment. From this initial sample, 4 participants were excluded because they had made too few “Buy” or “Not Buy” choices (<10% from all possible choices), data from two participants were discarded because of experimenter error during the study, data from one participant was lost because of a technical problem and data from one participant had excessive EEG artifact (66% of EEG data epochs had a peak-to-peak amplitude >100μV). The final sample had 32 participants (14 males, mean age: 23.3, SD: 2.8). They were all English-speaking; they had a normal or corrected vision; they did not report any history of psychiatric or neurological problems and they all reported to be right-handed. The Ethics committee of Durham University’s Psychology department approved the study and all participants signed an informed consent before taking part in the experiment.

### Stimuli

For this experiment, we used digital images of 120 products selected from shops and online retailers known to be used by Durham University students. The set of products included a large variety of items, such as electronics, food, drinks, sports equipment, home decoration, and others. Market prices estimated as the mean of the price of each product in two outlets ranged from £4 to £20 (Mean = 11.1, *SD* = 4.7). Products were randomly allocated to different conditions across participants to avoid potential confounds between market price and the experimental manipulation and other stimulus-specific factors, such as desirability. Manipulation checks about stimulus-specific factors are described in the Behavioural results section.

### Behavioural paradigm

Participants sat in a comfortable armchair at approximately 70 cm from a 19” Sony Trinitron CRT monitor with a refresh rate of 85Hz on which the stimuli were displayed. E-Prime 2.0 (Psychology Software Tools, Pittsburgh, PA) was used to display the stimuli on the screen. Participants were asked to perform a virtual shopping task while their scalp EEG was recorded. In this task, each participant was shown a sequence of 120 products on a computer screen. Each product was shown twice resulting in 240 trials. In each trial, participants were first shown a product, next they had to provide an estimate of the product’s market price and they were subsequently offered to buy or not this product at an offer price set by a computer program. Participants could decide to “buy” or not this product from of a virtual allocation of £35, which was reset for every trial. At the end of the experiment, a computer program randomly selected one of the products for which a “buy” decision had been made. The chosen product was later shipped to the participant, and cash “savings” corresponding to the initial allocation (£35) minus the price of the chosen product was paid to the participant. This approach was used to maximize the realism of the shopping task because participants had a real chance of walking out with one of the “purchased” products, and because cash savings are an inherent part of price-based shopping behaviour. All of these aspects of the behavioural paradigm were explicitly explained to the participant before the start of the experiment. The procedure of each trial is described in more details in Fig 1.

In half of the trials, the offer price at stage #9 was set to deviate by a large extent (75%) from the price estimate provided in stage #3. This level of price deviation was chosen because it has been shown previously to be optimal in modulating buying decisions in a virtual shopping task [3], and was used to induce a *large* prediction error. In the other half of the trials, offer prices were set to closely approximate participants’ estimates in order to induce a *small* prediction error using an average deviation of 8% relative to the price estimate provided in stage #3. In this case, we had to address two constraints. First, the offer price could not be equivalent to the estimate, otherwise a surprise reaction could be triggered by the exact match between the estimate and the offer. Second, a small fixed difference between the estimate and the offer could create the awareness that the offer price was a predictable calculation from the participant’s estimate, which could potentially lead to expectation effects. In order to minimize this possibility, offer prices deviated randomly from 1% to 15% relative to the estimate (average deviation: 8%) in this condition. The Behavioural results section provides manipulation check data showing that this approach was successful in modulating buying behaviour and the pattern of FRN results rules out potential confounds linked to this methodological approach: If FRN results were driven by a difference in the fixed vs. distribution-based nature of the deviations (rather than a difference in positive prediction error), then all large PE trials should be differentiated from small PE trials in terms of FRN amplitude. However, this prediction is contradicted by the absence of a reliable OL-OS contrast in the FRN data, which invalidates this alternative explanation.

These large or small prediction errors could be positive (cheaper than estimates) or negative (more expensive than estimates), resulting in four experimental conditions: Underpriced-Large (UL), Underpriced-Small (US), Overpriced-Large (OL), Overpriced-Small (OS). The number of trials per condition was equal (60), and the order of trials pertaining to any of the four conditions was fully randomized across the experiment. The repetition of the products across the experiment was used to maximize the number of trials thus increasing signal-to-noise ratio. A repeated trial did not necessarily belong to the same price deviation condition as the first (i.e. the randomization was full across all trials regardless of repetition), resulting in different offer prices, which minimized expectation effects. In addition, the allocation of specific product pictures to each condition was randomly varied between participants to avoid confounds between the experimental manipulation and product-specific parameters.

Finally, in order to minimize strategies based on buying only a small subset of products, participants lost money on their final cash savings if they did not buy a minimum threshold of products, according to a schedule previously explained to participants. If fewer than 25 products were bought, then £10 were subtracted from the final pay. If the amount of products bought ranged from 25 to 48, £5 were subtracted, and £1 was subtracted for the 49–72 range. If participants bought more than 72 products, no penalties were applied. All of this was explained to the participants before the experiment. At the end of the experiment, participants were again shown all the products offered during the experiment (without prices) and had to rate them on a 5-point scale of desirability (“how much do you want this product?” 1 = I don’t want it at all; 3 = I want it a bit; 5 = I want it very much); and a 5-point scale of Familiarity (“How familiar are you with this product”? 1 = Not familiar at all; 3 = Somewhat familiar; 5 = Extremely familiar). The experiment lasted approximately 2 hours.

### Electrophysiological data recording and pre-processing

**Fig 2:**
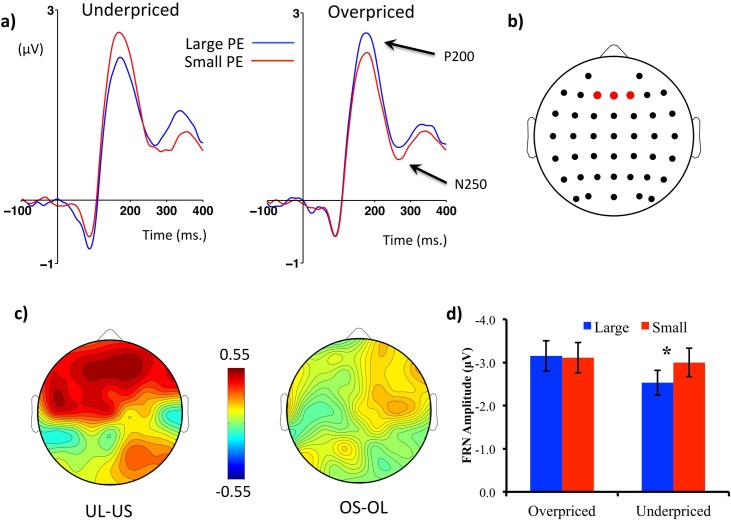
FRN as a function of price expectation violations. (a) ERP waveforms from a cluster of frontal electrodes time-locked to the offer price (stage #9) separated according to price valence (overpriced vs. underpriced) and price prediction error (large vs. small). Amplitude in microvolts (μV) is on the y axis and time in milliseconds is on the x axis. The arrows indicate the positive and negative peaks used to quantify the FRN (See Methods section) (b) Cluster of frontal electrodes (F1, Fz, F2) used to compute FRN components. (c) Scalp maps plotting contrasts of FRN peak to peak amplitudes between large and small prediction errors separated for underpriced and overpriced conditions, arranged so that more positive values reflect a greater positive valence (UL-US and OS-OL). The colour bar represents maxima and minima (μV). (d) Bar chart plotting peak-to-peak FRN amplitudes (μV) as a function of price valence and price prediction error. * *p* < .05.

Each participant’s scalp electroencephalogram (EEG) was recorded using 64 Ag/AgCl electrodes embedded in “Waveguard” purpose-made caps (the electrode layout is described in Fig 2B) and an “ASALAB” amplifier (both manufactured by ANT Neuro, Enschede, Netherlands). EEG data was recorded at a rate of 512Hz (DC-138 Hz bandwidth) and an impedance < 10kΩ, using a common average reference, which was digitally converted to an average mastoids reference. EEG data was pre-processed using EEGLAB version 10.256b [41] and ERPLAB version 4.023 [42]. Data was filtered offline (0.1-30Hz), segmented into epochs between 200 ms before and 1500 ms after the onset of the “offer price” screen (stage #9 of Fig 1) and baseline corrected. An average of 3.1 channels were found to be artifactual and were interpolated either through spherical spline interpolation or nearest-neighbour replacement.

EEG artifacts were attenuated following a multi-stage approach consistent with previous research [43–45]. First, Independent Component Analysis (ICA) was performed on epoched data using the "Infomax" ICA decomposition method implemented in the "*runica*()" function of EEGLAB [41]. This analysis enabled us to identify and remove components accounting for large ocular artifacts (eyeblinks and lateral eye movements) as well as components accounting for other large artifacts (e.g. muscle tension) following the guidelines of Jung and colleagues [46]. Second, in order to minimize artifacts not captured by ICA, we rejected data epochs that had a difference between the maximum and minimum voltage amplitudes exceeding 100 μV. In line with recommended practice [47], none of the 32 participants had more than a third of trials rejected by this technique. Data from one additional participant was discarded because 66% of their data epochs were rejected (see “Participants” section). Within the final sample of 32 participants, an average of 3.76% (*SD* = 6.0) of trials were removed per participant following this technique (max: 24.8%). Third, we ran a second ICA decomposition in order to identify and remove residual artifacts not captured in the initial steps.

In order to test whether the FRN was sensitive to price evaluation effects, ERP waveforms time-locked to the onset of the screen showing the products' offer price (stage #9) were created by averaging EEG epochs separately for UL, US, OL and OS conditions. The mean number of artifact-free trials per condition was: 51.3, 52.6, 50.9 and 52.2, respectively. None of the participants had less than 34 artifact-free trials in any of these conditions. In order to test for a potential “Buy/No-Buy” (BNB) effect on the FRN, ERPs were also separated by whether the product was subsequently purchased or not in stage #10 of the procedure. Mean artifact-free trials were 117.2 (min: 50) and 89.5 (min: 21) for “Buys” and “No-Buys”, respectively.

### ERP quantification

#### FRN

We focused on the Feedback-Related Negativity (FRN), which is a negativity peaking approximately between 200 and 350 ms in fronto-central electrodes [10]. Although the methods used to quantify the FRN can vary considerably in the literature, a growing trend suggests that the optimal way of quantifying this component involves computing peak-to-peak scores on individual feedback-locked ERP waveforms, in which a positive P200 peak is subtracted from a negative peak around ~250 ms [18,34,48–55]. This approach is thought to be better than using an absolute negative N200 peak or mean amplitude as it addresses potential biases relative to differences in the absolute amplitude of the onset of the FRN, which is typically located in a positive P200 peak that precedes it [18,48,49]. More generally, peak-to-peak scoring methods are typically used to minimize biases in the quantification of temporally overlapping components [49,56,57]. In addition, using individual waveforms instead of difference waveforms is recommended when testing hypotheses that involve comparing ERPs of the same valence [49,50]. Nevertheless, in order to compare with previous FRN literature we also computed difference waveforms, as explained hereafter.

We therefore computed scores in which the most positive local peak in a 150–220 ms window was subtracted from the most negative local peak in a 180–350 ms window. These time windows were drawn both from a vast literature on the FRN, P200 and N200 [10,19,49] and from a careful inspection of the timings of these components in the present data. Following previous research on the FRN, we focused our statistical analyses on a pre-defined scalp area around the Fz electrode [18,58,59]. We computed an average of three electrodes around this site (F1, Fz, F2, see Fig 2B) and all our FRN analyses were based on this cluster’s data. Pooling together single data from neighboring electrodes in a cluster is often recommended as it improves the stability of ERP data and attenuates familywise statistical errors [60–62].

We then ran an Analysis of Variance (ANOVA) on these peak-to-peak scores including the factors of Valence (Underpriced vs. Overpriced) and Prediction Error Size (Large vs. Small), followed by planned contrasts (UL-US and OL-OS) if an interaction was observed. Effects at *p* ≤ .05 were considered significant. In order to test the BNB effect, we also ran a simple F pairwise contrast to test if FRN peak-to-peak scores were different according to whether the product was subsequently purchased or not. Next, we examined if the BNB effect was driven by an influence of UL trials on FRN amplitude (see Results section). For this goal, we re-computed ERP waveforms time-locked to the “offer price” screen according to whether the product was subsequently purchased or not while removing UL trials in the overall computation. We then carried out a directional F contrast testing if FRN peak-to-peak scores were more positive for “Buy” than for “No-Buy” trials. As a measure of control, we also performed the same procedure while removing OL instead of UL trials. One participant was excluded after removing UL and 3 participants after removing OL as they did not have enough Buy and/or No-Buy artifact-free trials for a reliable analysis. We hypothesized that if UL trials were driving the BNB effect on the FRN, then this effect should be reduced or cancelled if UL trials were omitted, compared to a situation in which these trials were included in the analysis. Although the removal/inclusion of UL trials can help evaluate the impact of this specific group of trials on the BNB effect, this analysis should not be considered as a formal mediation test and any potential results have to be understood as tentative evidence of the role of PPE in buying decisions.

#### P300

**Fig 3:**
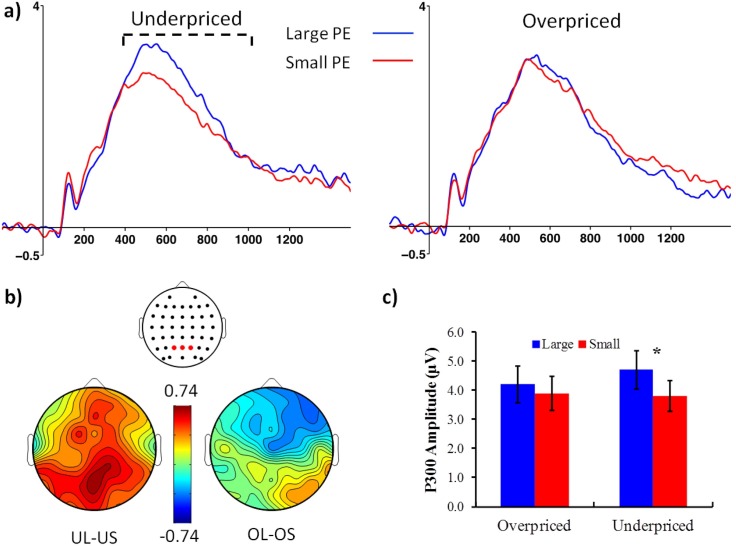
Feedback-Related P300 components. (a) ERP waveforms from a cluster of parietal electrodes time-locked to the offer price (stage #9) separated according to price valence (overpriced vs. underpriced) and price prediction error (large vs. small). The y axis shows amplitude in microvolts (μV) and x axis shows time in milliseconds. The dashed line indicates the time window used to quantify the P300. (b) Scalp maps showing P300 contrasts between underpriced large and underpriced small trials (UL–US; left) and between overpriced large and overpriced small trials (OL–OS; right), alongside a description of the location of the electrode cluster used in this analysis. Colour bar represents maxima and minima (μV). (c) Bar chart plotting P300 amplitudes (μV) as a function of price valence and price prediction error. * *p* < .05.

While our main focus was on the FRN as a measure of prediction error, we also analyzed a positive potential that follows the FRN, which is often labeled as a Feedback-Related P300. This ERP tends to be predominantly centro-parietal and resembles a P3b. The neural generator of this ERP is uncertain, but there is evidence linking this component to positive prediction error [51], although it can be sensitive to other aspects of outcome processing, and its functional meaning is still relatively unclear [50]. Following previous research on late positivities [39,63,64] and a careful examination of the ERP waveforms of the present study, we quantified a Feedback-Related P300 time-locked to the “offer price” screen computing a peak-to-peak score in which the most negative peak found in a 100–300 ms time window is subtracted from the most positive local peak found in a 400–1000 ms window. We focused our statistical analyses on a cluster of P1, Pz and P2 electrodes (see Fig 3B). This is in line with previous literature that typically locates the Feedback-Related P300 in the Pz area [18,58]. An ANOVA similar to the one used for FRN scores was computed. To mirror the analyses performed on the FRN, we also tested a BNB effect on the Feedback-Related P300, but this effect was not significant.

#### P2, P3a and FRN difference waveforms

Further, we also examined if two components known to partially overlap with the FRN were affected by our manipulation–the P2 and P3a components. The P2 was calculated as a peak-to-peak difference in which the local negative peak in a 50-130-ms window was subtracted from the local positive peak in the 150-220-ms window. The P3a was calculated as a peak to peak difference between a local positive peak in a 320-450-ms window [65,66] and a local N250 negative peak in the 180-350-ms window, similar to the one used for the FRN analysis. For both components, analyses focused on a cluster of frontal electrodes as the one described in Fig 2C. None of the key contrasts (UL-US, OL-OS, BNB) turned out to be statistically significant for these components and therefore they will not be discussed further. Finally, given that the FRN is often presented in difference waveforms, we also subtracted overpriced ERPs from underpriced ERPs for data relative to the cluster of 3 frontal electrodes used for all other FRN analyses separately for large and small prediction error conditions. This "gain-loss" difference waveform is the converse of the more classical "loss-gain" subtraction typically used in FRN data, but it conforms to more recent studies interpreting the FRN as a "reward positivity" signal [30] and aims to better isolate the positive deflection associated to reward feedbacks. Similarly to previous research using difference waveforms [67], we computed peak to peak scores for the two difference waveforms, taking the same time windows used to quantify the FRN in individual waveforms. However, in this case the N2 peak was subtracted from the P2 peak.

## Results

### Behavioural results

#### Buying rate

**Fig 4:**
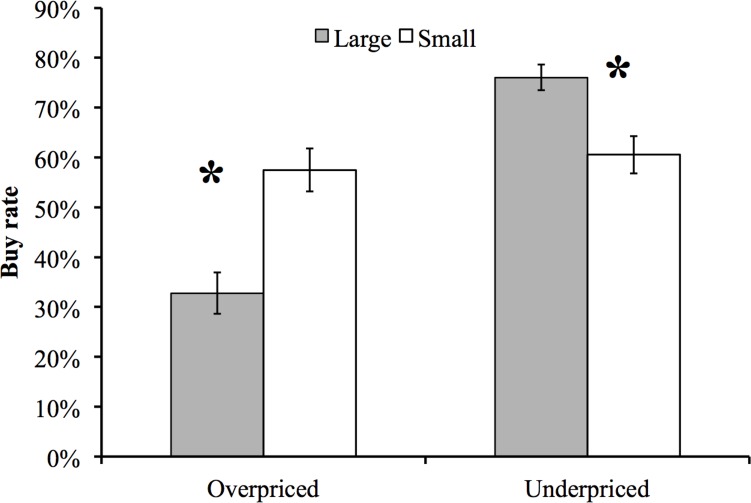
Buying behaviour. Buying rate (% of total valid trials) as a function of price valence (overpriced vs. underpriced) and price prediction error (large vs. small). * *p* < .05.

We analyzed behavioural results through a classical ANOVA testing the effects of Valence (Underpriced vs. Overpriced) and Prediction Error (Large vs. Small) on the rate of “Buy” responses. Not surprisingly, we found that participants bought more underpriced than overpriced products (main effect of valence, *F*(1,31) = 62.07, *p* < .000001, *η*_*p*_² = .67) and the main effect of Prediction Error did not reach significance levels *F*(1,31) = 2.98, *p* = .09, *η*_*p*_² = .09. We found an interaction, *F*(1,31) = 66.74, *p* = .000001, *η*_*p*_² = .68, driven by more buys in UL compared to US, *F*(1,32) = 19.76, *p* < .001, *η*_*p*_² = .39, and fewer buys in OL than OS, *F*(1,31) = 43.40, *p* = .000001, *η*_*p*_² = .58. These results confirm that our experimental manipulation had reliable and expected effects on buying decisions (see Fig 4).

#### Multilevel logistic regression analysis of buying decisions

Desirability, familiarity and market price were equated across UL, US, OL and OS conditions thanks to the full randomization of products and trials, and pairwise comparisons between these conditions did not reach significance levels for any of these variables (Desirability mean scores: 2.7, 2.6, 2.6, 2.7; Familiarity: 3.3, 3.2, 3.2, 3.3; Market Price: 11.1, 11.2, 11.2, 11.2, respectively for UL, US, OL and OS). Not surprisingly, mean cash “savings”were higher for UL and lower for OL (UL: 32.8; US: 26.7, OS: 25.3; OL: 19.3), and desirability tended to be higher for products linked to “Buy” compared to “No-Buy” trials (*Ms* = 3.2, 2.1; *SDs* = 1.2, 1.1).

In order to examine more closely the potential effects of these trial and product-specific factors on buying decisions, we used a generalized linear mixed models approach in order to test a multilevel logistic model in which the outcome variable was a binary factor coding whether a product had been purchased or not (1 or 0) in each trial. This approach enabled us to examine the determinants of buying decisions at the trial level while taking into account potential between-subjects variability. Predictors were self-reports of desirability and familiarity, product market price and cash savings (35 minus the offer price). Following the interaction observed in the ANOVA analysis, we also entered two binary predictors coding for the effects of UL and OL trials. Specifically, the UL predictor was a binary variable coding for the difference between UL and other trial types observed in the averaged ERP analysis (1 = UL, 0 = all other trials), and the OL variable coded for the difference between OL and other trials. The total number of trials considered in this analysis was high (*N* = 6612), which can inflate classical significance values. Therefore, we report hereafter Odds Ratios (*OR*) as an estimate of effect size. It has been proposed that an OR of 1.5 corresponds to a small effect size, an OR of 2.5 is a medium effect size and an effect of 4 is a large effect size [68].

Consistent with our expectations, we found that desirability strongly predicted “Buy” decisions (*b* = 0.94, *t* = 29.9, *p* < .001, *OR* = 2.6). Importantly, we found that both UL and OL had a unique effect on buying decision, while controlling for all the other factors: UL significantly predicted a robust increase in “Buy” choices (*b* = 0.98, *t* = 9.8, *p* < .001, *OR* = 2.7) and OL significantly predicted an increase in “No-Buy” choices (*b* = –1.5, *t* = –14.9, *p* < .001, OR = 4.5). Familiarity predicted a very small increase in “Buy” choices (*b* = 0.13, *t* = 4.6, *p* < .001, *OR* = 1.14), and although the effect of the product market price yielded a *p*-value below the .05 level, the *OR* indicates that the effect size is negligible (*b* = 0.02, *t* = 2.3, *p* = .002, *OR* = 1.03). Similarly, OR levels indicated that potential cash savings did not have a noticeable effect on participants’ behaviour (*b* = 0.02, *t* = 1.9, *p* = 0.06, *OR* = 1.02). These results indicate that the experimental manipulation of over- and underpricing had an effect on buying decisions over and above trial- and product-specific factors. Given that cash savings were directly linked to offer prices, these results also indicate that absolute offer prices and market prices did not drive participant’s behaviour, whereas the violations of price expectations did.

#### Response time

We conducted a repeated-measures ANOVA testing the effects of Valence, Prediction Error, and BNB (Buy vs. No-Buy) on mean response time to “Buy” or “No-Buy” choices (stage #10 of Fig 1). We found that participants responded to underpriced products (*M* = 767.33, *SD* = 343.42) marginally faster than to overpriced products (*M* = 874.43, *SD* = 596.18), *F*(1,31) = 3.90, *p* = .06, *η*_*p*_² = .11. We also observed a Valence × BNB interaction, *F*(1,31) = 8.41, *p* = .007, *η*_*p*_² = .21. This interaction was driven by the fact that participants were faster for “Buy” (*M* = 738.45, *SD* = 388.70) than “No-Buy” choices (*M* = 796.21, *SD* = 312.17) for underpriced products, *F*(1,31) = 4.21, *p* = .05, *η*_*p*_² = .12. This difference did not reach significance levels for overpriced products (Buy: *M* = 920.70, *SD* = 649.23; No-Buy: *M* = 828.16, *SD* = 575.49), *F*(1,31) = 3.28, *p* = .08, *η*_*p*_² = .10.However, these results were qualified by a higher-order 3-way interaction, *F*(1,31) = 8.15, *p* = .008, *η*_*p*_² = .21. To decompose this interaction, we conducted Valence x BNB ANOVAs for Large Prediction Error and Small Prediction Error separately. The 2-way interaction was not significant for Small Prediction Error, *F* < 1. However, it was significant for Large Prediction Error, *F*(1,31) = 12.02, *p* = .002, *η*_*p*_² = .28. This interaction was caused by faster responses for “Buy” (*M* = 690.36, *SD* = 319.83) than “No-Buy” (*M* = 795.18, *SD* = 334.42) decisions in UL, *F*(1, 31) = 6.82, *p* = .014, *η*_p_^2^ = .18. On the other hand, in OL, responses were faster for “No-Buy” (*M* = 748.03, *SD* = 291.18) than “Buy” (*M* = 948.84, *SD* = 563.53) decisions, *F*(1, 31) = 6.45, *p* = .016, *η*_p_^2^ = .17. These findings suggest that “buy” decisions were facilitated in the UL condition whereas “No-Buy” decisions were facilitated in the OL condition.

### Brain potentials

**Fig 5:**
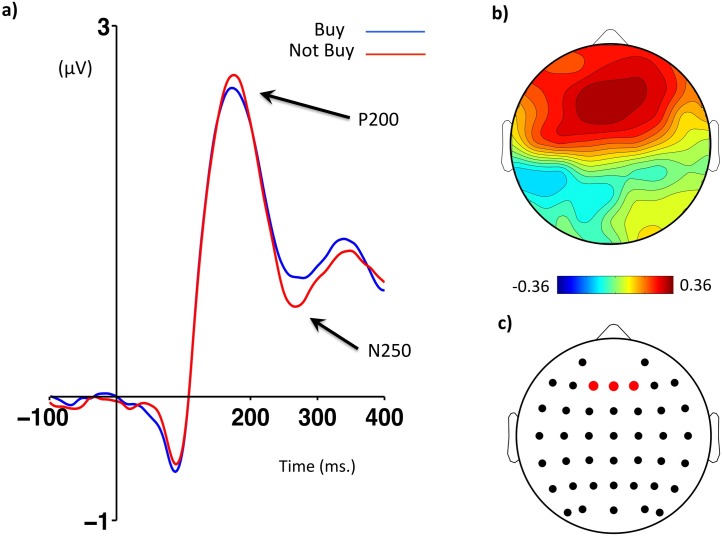
FRNs as a function of buying decisions. (a) Waveforms from a cluster of frontal electrodes time-locked to the offer price (stage #9) separated by whether the product was subsequently purchased (“Buy”) or not (“Not Buy”) in stage #10. Amplitude in microvolts (μV) is shown on the y axis and time in milliseconds is shown on the x axis. The arrows indicate the positive and negative peaks used to quantify the FRN (See Methods section). (b) Scalp maps showing a contrast between “Buy” and “Not Buy” related FRNs. Color bar represents maxima and minima (μV). (c) Cluster of frontal electrodes (F1, Fz, F2) used to compute FRNs.

Fig 2A shows a large negative deflection with a P200 onset and a negative peak around ~270 ms. The peak-to-peak amplitude of this deflection seems to be smaller for UL compared to US trials, whereas the amplitude of this signal seems equivalent between OL and OS (Fig 2D). Scalp maps (Fig 2C) show that this amplitude reduction (plotted as a positive value in Fig 2C) is predominantly frontal, in line with previous research on the FRN [10,50,69]. The same figure also shows that differences between OS and OL are much smaller compared to UL-US contrasts. These results seem to confirm the hypothesis that FRN amplitude would be more positive (by a reduction of its negativity) for offers that are underpriced by a large amount. In addition, Fig 5 suggests that the FRN measured at the time of an offer is able to differentiate products that are subsequently purchased or not, and the topography of this effect also conforms to the typical topography of the FRN. Fig 3A shows a larger P300 positivity for UL compared to US trials, and this effect seems predominantly parietal (Fig 3B). Similar to the FRN data, OL-OS contrasts seem to yield much lower scores than UL-US contrasts across the whole scalp (Fig 3B).

Statistical analyses have supported these observations. Analyses on peak-to-peak estimates of the FRN revealed a main effect of Valence, [*F*(1, 31) = 6.8, *p* = .01, *η*_*p*_² = .18], indicating an overall larger negativity for overpriced compared to underpriced offers (*M* = –3.1, *SE* = .34; *M* = –2.8, *SE* = .30); and the main effect of Prediction Error did not reach significance levels [*F*(1, 31) = 3.1, *p* = .09, *η*_*p*_² = .09]. Consistent with our hypotheses, we observed a significant Valence × Prediction Error interaction [*F*(1, 31) = 6.2, *p* = .02, *η*_*p*_² = .17]. This interaction was driven by a large difference between UL and US, *F*(1,31) = 12.8, *p* = .001, *η*_*p*_² = .29, whereas the OL-OS contrast was not significant, *F* < 1. These results indicate that FRN amplitudes are more positive for offers that are underpriced by a large amount, as shown in Fig 2. We also verified that the UL-OL contrast was significant, *F*(1,31) = 10.8, *p* = .003, *η*_*p*_² = .26, contrary to the OS-US, *F* < 1, which is consistent with a vast literature indicating that the effect of valence on the FRN is stronger for unexpected outcomes [10]. In order to provide a comparison with FRN studies that use difference waveforms, we also examined difference waveforms in which overpriced ERPs were subtracted from underpriced ERPs separately for large and small prediction errors (see Methods section for more details). As seen in the S1 Fig, a clear positive N2-P2 deflection is visible for large prediction errors in the FRN time window (peaking at approximately 330 ms), whereas there is virtually no discernable positive deflection for the “small” condition. These results were confirmed by a significant difference between peak-to-peak scores for the two waveforms [*t*(31) = 2.9, *p* = .007] confirming that the FRN had a larger amplitude for large than for small prediction errors. This result confirms that our FRN effects are compatible with previous data using difference waveforms [70].

To examine a potential Buy/No-Buy (BNB) effect on the FRN, we compared the FRN for offers leading to subsequent purchases compared to offers followed by “No-Buy” choices. The contrast was significant, [*F*(1, 31) = 6.3, *p* = .02, *η*_*p*_² = .17], indicating that the FRN was significantly larger for “No-Buy” compared to “Buy” choices (*M*s = –2.94, –2.5, *SE*s: 0.32, 0.28). In other words, “Buy” decisions were preceded by a more positive FRN amplitude than “No-Buy”decisions. Consistent with our hypothesis, the difference between “Buy” and “No-Buy” FRNs was flattened after removing UL trials, (difference = –.03, *SE* = 0.18; directional *t*-test: *p* = .42, *ns*.), whereas the effect remained reliable when only OL trials were omitted (difference = 0.40, *SE* = .20, directional *t*-test: *p* = .03). This specific finding tentatively suggests that the FRN predicts subsequent buying decisions if these are driven by a positive surprise regarding the price of the product.

Analyses on the P300 revealed no main effect of Valence [*F*(1, 31) = 1.1, *p* = .29, *η*_*p*_² = .03] but found a main effect of Prediction Error [*F*(1, 31) = 9.8, *p* = .004, *η*_*p*_² = .13], indicating that overall P300 amplitude was more positive for large than for small prediction errors (*M*s = 4.5, 3.9, *SE*s: .64, .55); and a Valence × Prediction Error interaction [*F*(1, 31) = 5.1, *p* = .03, *η*_*p*_² = .14]. This interaction was caused by a large difference between UL and US, [*F*(1,31) = 16.8, *p* = .0003, *η*_*p*_² = .35], whereas the OL-OS contrast was not significant, [*F*(1,31) = 1.1, *p* = .31, *η*_*p*_² = .03]. The contrast testing if P300 amplitudes varied according to subsequent “Buy”or “No-Buy” choices was not significant [*F*(1,31) = 2.5, *p* = .12, *η*_*p*_² = .07; *M*s = 4.2, 3.9, *SE*s = .62, .59]. In addition, we also verified that FRN and P300 results were site-specific, as explained in S1 Results.

## Discussion

We found here that a well-known neural index of prediction error computation, the FRN brain potential, was related to two fundamental aspects of consumer behaviour: Price evaluation and buying decisions. More specifically, we first found that FRN amplitude was more positive when prices were substantially cheaper than participants’ expectations. Second, we found that FRN amplitude measured during price evaluation was more positive when followed by a “Buy” rather than by a “No-Buy” decision. Interestingly, this effect disappeared when UL trials were omitted, tentatively suggesting that it was driven by an effect of surprise to unexpectedly cheap prices. Finally, we also found an increase in P300 positivity when offered prices were cheaper than expected.

A potential explanation for our results would posit that a more positive FRN amplitude for UL trials would reflect positive prediction error (PPE) signals caused by a “surprise” reaction to unexpectedly cheap prices. This explanation would be consistent with previous research showing that the classical FRN effect (a distinction between negative and positive outcomes) is mainly driven by the FRN sensitivity to unexpected positive outcomes, similar to what we observed in the current study [10,29,31,33,71]. This effect of “reward positivity” [30] is explained in one of the main theories of the FRN, the “Reinforcement Learning–Error Related Negativity” theory [11,31] by a mechanism of inhibition of ACC neurons by dopaminergic projections from subcortical structures when PPEs are detected [31]. This model is compatible with existing evidence of the relationship between PPE and the dopaminergic network [72]. As mentioned in the Introduction, it has to be noted that a few FRN studies have shown that large PPEs can in certain cases be related to a more negative FRN deflection compared to small PPEs [34,35,50,73], which goes contrary to the “Reward Positivity” explanation. As discussed in the Introduction, the origin of this discrepancy is still unresolved but it might be caused by the existence of distinct groups of ACC neurons that react in different ways to positive surprise [37]. Nevertheless, our results are compatible with a vast body of literature showing a more positive FRN amplitude for unexpected positive outcomes [10,30,33].

This explanation assumes that consumers form price expectations by previous exposure to varying prices of a same product in a process similar to the formation of predictions described by RL theory. This learning process would create a representation of the monetary value of this product, which has been described as “price expectation”, “reference price” or “fair price” in the consumer behaviour literature [1]. When an instance of this product is encountered with a price markedly cheaper than the learned expected price, then subcortical brain structures such as the VTA [10] or the basal ganglia [74] would interpret this event as a PPE. Consequently, phasic dopaminergic signals from these structures would inhibit ACC neurons, resulting in a more positive FRN amplitude [18].The ACC is known to be activated when the need to effectuate goal-relevant action is detected [75,76], and this structure is thought to be able to bias decision-making through its connections with other cognitive and motor brain systems [77,78]. Therefore, its modulation by price variations is very likely to lead to behavioural consequences in terms of buying decisions, as suggested by our observed BNB effect on FRN amplitude.

This explanation is also consistent with previous research using functional magnetic resonance imaging (fMRI) reporting an association between increased activity in the medial prefrontal cortex (MPFC) and price-related consumer behaviour [3,6,7,79]. MPFC areas, and in particular the ACC, are thought to be involved in PE computation. However, it has to be noted that a particular brain area is often made of different groups of neurons that can perform different types of computations [80,81]. This is particularly true for the MPFC, which has been associated to many distinct psychological processes [13–17,32,78], and thus its activation cannot establish by itself the recruitment of PE-related processes. Given that the FRN is widely seen as an index of PE computation, our results suggest that these processes may have been involved in previous findings of MPFC activity during shopping tasks.

Although we did not have specific hypotheses about the P300, we found that its amplitude was more positive for UL than US trials. Nevertheless, it did not reliably predict buying decisions. The P300 is mostly related to the mobilization of attentional resources [39,64] and in the decision-making literature, this component tends to be larger for positive than negative outcomes [40,82]. Consequently, the P300 seems to reflect a preference of attentional systems towards positive prediction errors in decision-making environments [18]. The lack of links with buying decisions suggests that this attentional response may not necessarily be linked to the fulfillment of a value-related goal. Alternatively, a link between attention to price stimuli and buying decisions might depend on parameters not investigated in this study. For instance, a recent study [83] found a relation between the P300 and buying decisions that varied according to individual differences (e.g., trait anxiety).

A number of alternative explanations and caveats about our interpretations need to be discussed. First, it could be argued that our FRN results are caused by changes in product desirability, rather than by an effect of PPE. However, desirability scores were equated across the four key UL, US, OL and OS conditions through our randomization procedures and thus price difference effects on the FRN cannot be accounted for by differences in desirability. Furthermore, the BNB effect on the FRN seems to be accounted for by UL trials, which invalidates a desirability-based explanation of the BNB effect, given that the UL condition does not differ from other conditions in terms of desirability scores. Second, as explained in the Methods section, the fact that small PEs were obtained through distribution-based deviations and that large PEs were obtained through fixed-percentage deviations could create a potential confound. Therefore, one could argue that PE size effects can actually be merely explained by a “fixed vs. distribution” effect on FRN amplitude. Although we do acknowledge that this explanation is possible, the nature of the FRN results makes it highly unlikely: If FRN results were affected by a “fixed vs. distribution” effect, then all large PE conditions should be different from all small PE conditions in a uniform way. However, this prediction is invalidated by the absence of reliable OL-OS differences in the FRN data, which strongly contradicts this alternative explanation. Third, it could be posited that the FRN was modulated by an affective reaction to the anticipation of potential cash savings obtained by buying cheap products, regardless of our under- and over-pricing manipulation. However, this explanation would predict a graded difference in FRN amplitude that mirrors potential cash savings (OL<OS/US<UL). This prediction is disconfirmed by the absence of OL-OS differences in our FRN data, which renders unlikely an explanation based on affective reactions to the anticipation of savings. This explanation is further compromised by the finding that our behavioural analyses showed that the effects of potential cash savings on buying decisions was virtually non-existent, which suggests that participants’ behaviour was not driven by a “savings” strategy. Finally, the greater amount of buys in UL compared to other conditions is inherent to shopping behaviour, in which underpricing leads to more purchasing decisions if all other parameters are held constant. Therefore it is worthwhile to discuss in more details whether this could create important confounds. As indicated by our analysis of BNB effects after the removal of specific types of trials, this phenomenon seems to account for the BNB effects in that the BNB effect on FRN amplitude is probably explained by the effects of UL on FRN amplitude, which is perfectly consistent with our hypotheses. However, the greater amount of buys in the UL condition could also suggest that the effects of price deviations on ERPs could be confounded by differences in the frequencies of Buy/No-Buy decisions. The fact that buying decisions occur after the feedback (price offers) to which ERPs were time-locked limits the extent to which buying decisions could modulate the relationship between price deviation and the FRN/P300. However, one could argue that an “intention to acquire” could modulate neural activity at the time of price offers, above and beyond the effects of price deviations. However, a putative “intention to acquire” that would be independent from price manipulations is likely to have been captured by our desirability scales, which operationalized desirability as an intention to obtain/acquire the product (or "wanting"). An important aspect of these desirability scores is that they were equated across all price conditions (UL, US, OL, OS) and therefore it is unlikely that a price-independent “intention to acquire” could explain the FRN/P300 relationship with price deviations. Consequently, an “intention to acquire” effect at the stage of price offers would be possible only if this intention was fully driven by the manipulation of price deviations. However, this would lead to the implication that FRN/P300 effects are ultimately accounted for by price manipulations, which would be consistent with our main explanation. Furthermore, we also re-computed the key UL-US contrast in our FRN data only for trials followed by a "Buy" decision on 29 participants who had enough artifact-free trials for this comparison. If price deviation effects were accounted for by differences between Buy and No-Buy trials, then the key UL-US contrast should be reduced if only "Buy" trials were considered. We observed that this contrast remained significant [*F*(1, 28) = 10.5, *p* = .003, *η*_*p*_² = .27, see S2 Fig], and therefore, we found no evidence of a BNB confound in our manipulation of price deviations. However, we do acknowledge that future research will be needed to fully address this issue, in experiments where price deviations and intention to acquire are independently manipulated.

In conclusion, our results show that two brain potentials usually linked to unexpected outcomes–the FRN and the P300 are sensitive to the violation of price expectations in a virtual shopping task. Although we acknowledge that a number of alternative explanations can be advanced (e.g. affective reactions to cash savings, desirability, etc.), our results seem to better fit an explanation based on positive prediction error mechanisms. Future research will be needed to confirm these findings, but the current data opens new perspectives for the field of consumer neuroscience: First, this study suggests that groups of neurons coding positive prediction errors play a critical role in consumer decision-making, and thus electrophysiological biomarkers of reward processing [30] might potentially become useful tools to model consumer behaviour. Second, the link between well-known biomarkers of prediction error computation and key aspects of consumer decision-making suggests that RL theory has a strong potential to form neurobiologically grounded theoretical models of consumer behaviour. Third, this study focused on price expectations (or “fair price”), but future research could investigate if the FRN and P300 could predict willingness to pay (WTP), an important concept directly linked to buying intentions [84]. Previous research has already shown that WTP is linked to MPFC activity, which suggests that PE mechanisms may be involved [7]. Finally, the finding of a link between electrophysiological signals of prediction error and purchasing choice suggests that low-cost neuroscience techniques such as EEG and the Event-Related Potentials method (ERP) can serve as tools to better understand and predict consumer behaviour.

## Supporting Information

S1 Fig Difference waveforms for the FRN. Difference waveforms in which overpriced ERPs were subtracted from underpriced ERPs, separately for large and small prediction errors. The y axis shows amplitude in microvolts (μV) and x axis shows time in milliseconds. The circle and arrow indicate the positive peak of the difference waveform in the “Reward Positivity” time window.
(TIFF)

S2 Fig ERP waveforms for “Buy” trials. ERP waveforms from a cluster of frontal electrodes time-locked to the offer price (stage #9) separated according to UL and US trials. Only trials followed by "Buy" decisions are taken into account for this figure. Amplitude in microvolts (μV) is on the y axis and time in milliseconds is on the x axis.
(TIFF)

S1 Results Supplementary Results. File containing supplementary analyses.
(DOCX)
