# Supplementary figures and images for: The Feedback-Related Negativity and the P300 Brain Potential Are Sensitive to Price Expectation Violations in a Virtual Shopping Task
Source: PLoS One. 2016 Sep 22;11(9):e0163150. doi: 10.1371/journal.pone.0163150 (PMC5033321; doi:10.1371/journal.pone.0163150)

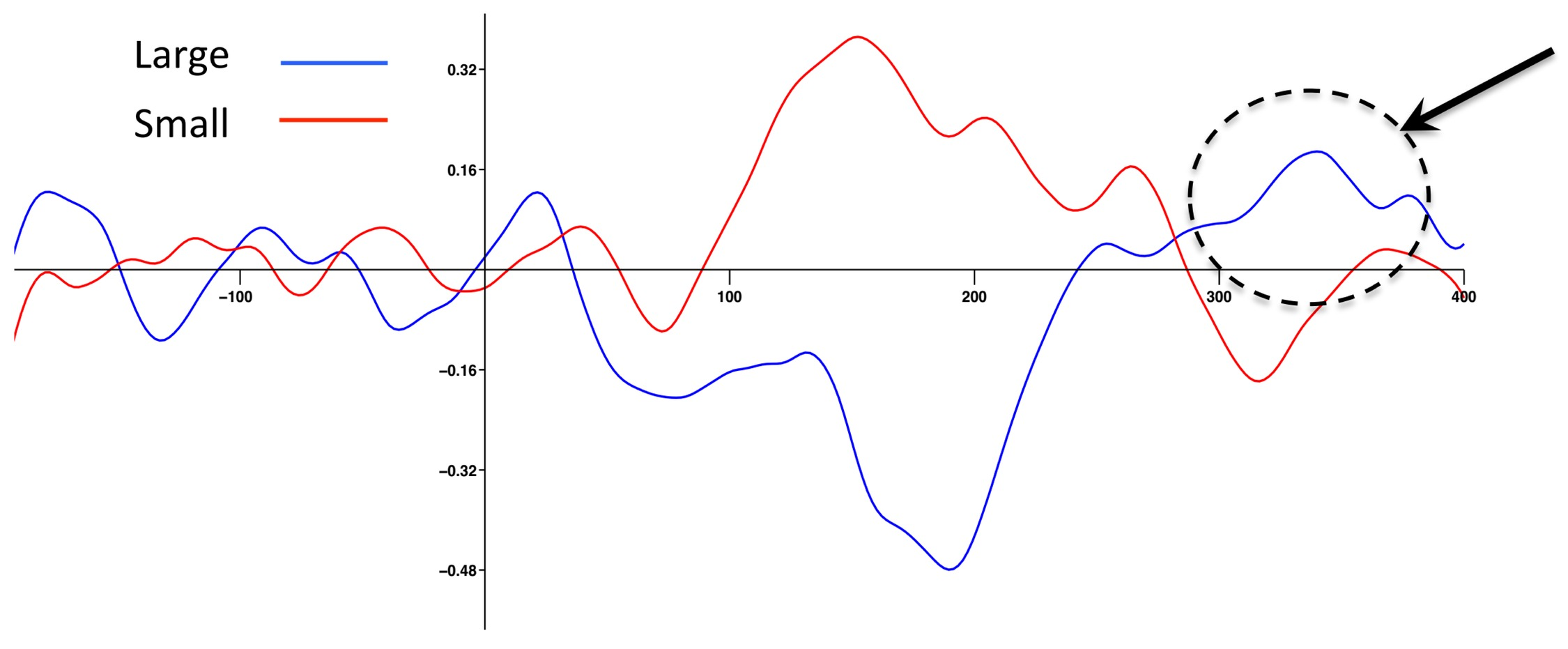

Supplement: S1 Fig — Difference waveforms in which overpriced ERPs were subtracted from underpriced ERPs, separately for large and small prediction errors. The y axis shows amplitude in microvolts (μV) and x axis shows time in milliseconds. The circle and arrow indicate the positive peak of the difference waveform in the “Reward Positivity” time window. (TIFF) [file pone.0163150.s001.tiff]

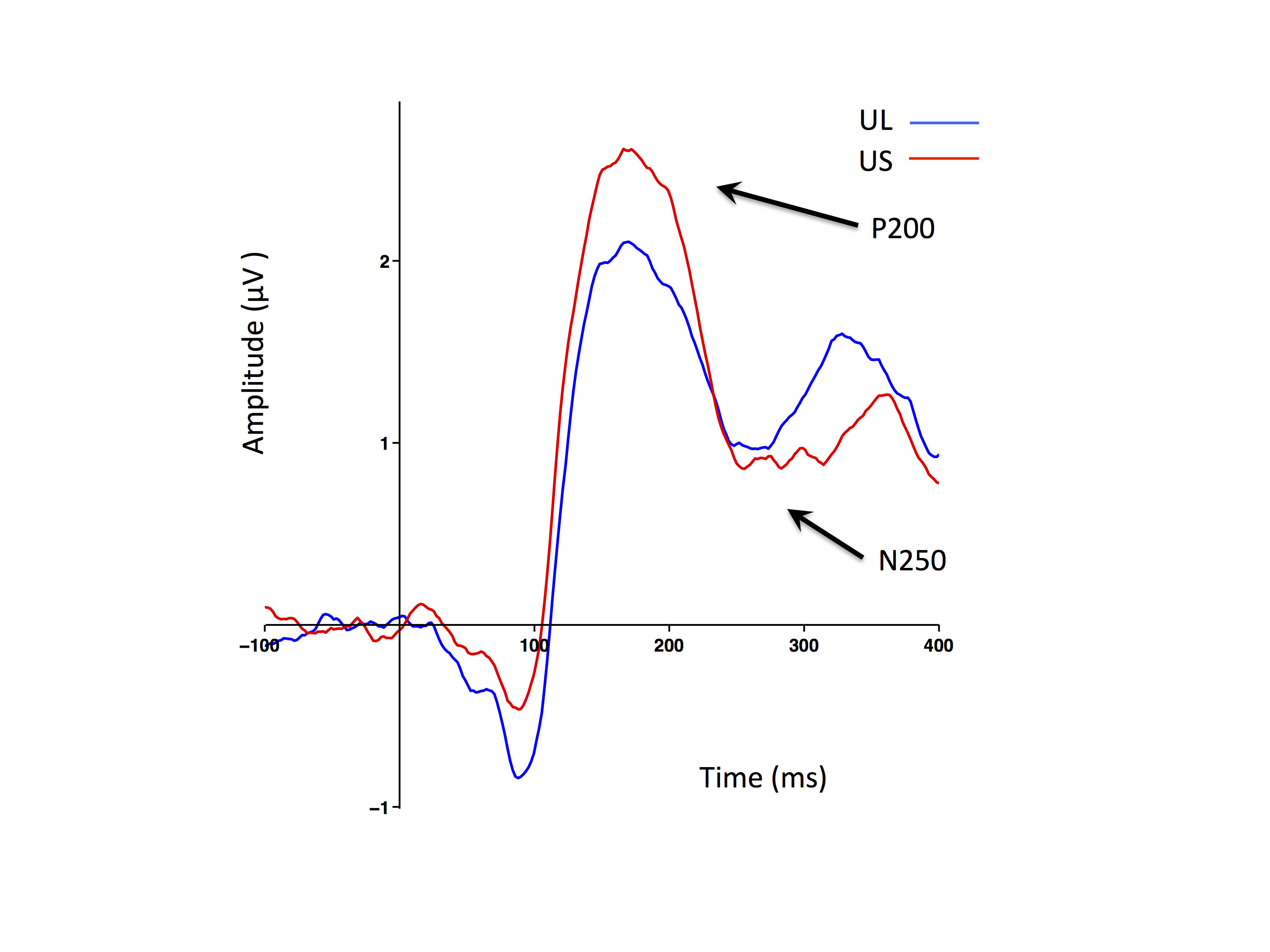

Supplement: S2 Fig — ERP waveforms from a cluster of frontal electrodes time-locked to the offer price (stage #9) separated according to UL and US trials. Only trials followed by "Buy" decisions are taken into account for this figure. Amplitude in microvolts (μV) is on the y axis and time in milliseconds is on the x axis. (TIFF) [file pone.0163150.s002.tiff]
