# Supplementary material for: The Feedback-Related Negativity and the P300 Brain Potential Are Sensitive to Price Expectation Violations in a Virtual Shopping Task
Source: PLoS One. 2016 Sep 22;11(9):e0163150. doi: 10.1371/journal.pone.0163150 (PMC5033321; doi:10.1371/journal.pone.0163150)
Supplement: S1 Results — File containing supplementary analyses. (DOCX) [file pone.0163150.s003.docx]

**Supplementary Results S1**

**Site specificity of the FRN and P300 effects**

Although a priori selections of electrode sites is a common approach to study the FRN, as well as other ERP effects (Luck, 2005), one could argue that our approach cannot establish if the FRN effects that we observed are specific to frontal sites or if they can be observed all throughout the scalp. Therefore, we computed peak-to-peak scores for the cluster of parietal electrodes (see Fig 3b), following the parameters used to compute these scores for the FRN, as described in the Methods section, and we ran a Prediction Error × Valence × Site (Frontal vs. Parietal) ANOVA. The 3-way interaction between these 3 factors was significant, *F*(1, 31) = 11.2, *p* =.002, *η_p_*² = .26. Subsidiary analyses showed that the Prediction Error × Valence interaction was not significant in parietal sites, *F*(1, 31) = 1.6, *p* = .21, *η_p_*² = .05, whereas it was significant in frontal sites, as shown in the main results section, *F*(1, 31) = 6.2, *p* = .02, *η_p_*² = .17. We applied the same logic to the BNB effect, and found a Site × BNB interaction, *F*(1, 31) = 4.5, *p* = .04, *η_p_*² = .12. The BNB effect was not significant in parietal sites (*F* < 1), whereas it was in frontal sites, as described in the previous section.

The same approach was applied to the P300 effect. We re-computed a P300 effect on frontal cluster data (see Figure 3b for the locations of the electrodes included in the cluster) and found a significant Prediction Error × Valence × Site interaction, *F*(1, 31) = 4.2, *p* < .05, *η_p_*² = .12, driven by the finding that the Prediction Error × Valence interaction was not significant in frontal sites, *F*(1, 31) = 1.8, *p* = .19, *η_p_*² = .05, whereas it was in parietal sites, as described in the previous section. Overall, these results confirm that FRN and P300 effects reported in the main results section are localized in specific scalp locations.
